# Age-specific Contributions to Height in Boys with Klinefelter Syndrome: Analysis of Growth Using the QEPS Model

**DOI:** 10.1210/jendso/bvaf206

**Published:** 2025-12-12

**Authors:** Anton Holmgren, Aimon Niklasson, Lars Gelander, Andreas F M Nierop, Aldina Pivodic, Anders Juul, Lise Aksglaede

**Affiliations:** Department of Pediatrics, Institute of Clinical Sciences, Sahlgrenska Academy, University of Gothenburg, 41685 Gothenburg, Sweden; Department of Pediatrics, Halland Hospital, 30185 Halmstad, Sweden; Department of Research and Development, Region Halland, Box 517, 301 80 Halmstad, Sweden; Department of Pediatrics, Institute of Clinical Sciences, Sahlgrenska Academy, University of Gothenburg, 41685 Gothenburg, Sweden; Department of Pediatrics, Institute of Clinical Sciences, Sahlgrenska Academy, University of Gothenburg, 41685 Gothenburg, Sweden; Department of Pediatrics, Institute of Clinical Sciences, Sahlgrenska Academy, University of Gothenburg, 41685 Gothenburg, Sweden; Muvara bv, Multivariate Analysis of Research Data, 2353 PH Leiderdorp, The Netherlands; APNC, Entreprenörsstråket 10, 431 53 Mölndal, Sweden; Department of Growth and Reproduction, Copenhagen University Hospital—Rigshospitalet, 2100 Copenhagen, Denmark; International Centre for Research and Research Training in Endocrine Disruption of Male Reproduction and Child Health, Copenhagen University Hospital—Rigshospitalet, 2100 Copenhagen, Denmark; Department of Clinical Medicine, Faculty of Health and Medical Sciences, University of Copenhagen, 2200 Copenhagen, Denmark; Department of Growth and Reproduction, Copenhagen University Hospital—Rigshospitalet, 2100 Copenhagen, Denmark; International Centre for Research and Research Training in Endocrine Disruption of Male Reproduction and Child Health, Copenhagen University Hospital—Rigshospitalet, 2100 Copenhagen, Denmark

**Keywords:** Klinefelter syndrome, growth patterns, QEPS growth model, TRT

## Abstract

**Context:**

Increased height is a characteristic of Klinefelter syndrome (KS). Detailed evaluation of growth patterns in boys and adolescents carrying a 47,XXY karyotype is lacking.

**Objective:**

To delineate detailed growth patterns during the different growth phases and their contribution to the increased adult height in boys with KS.

**Methods:**

Longitudinal data on growth from 55 boys with KS were compared with a reference from the GrowUp_1974_Gothenburg cohort using a statistical growth model using 4 mathematical functions: the QEPS model. A subgroup of 35 boys out of whom 34 were on testosterone replacement therapy (TRT) reached final height.

**Results:**

The infant growth period was shorter with a lower height gain (*Emax*, 63.3 vs 65.1 cm, *P* < .001) in boys with KS. The boys gained more height during the childhood growth phase, (*Qmax*, 110.9 vs 104.1 cm, *P* < .001), and onset of pubertal growth was earlier as compared with the reference (11.3 vs 11.8 years, *P* < .001). The total pubertal height gain was higher (32.9 vs 30.6 cm, *P* < .001) due to more basic growth; the specific pubertal growth was equal, resulting in a taller adult height (184.6 vs 180.5 cm, *P* < .001).

**Conclusion:**

The boys with KS exhibited a different growth pattern as compared with a healthy reference population, with less and shorter growth in infant life and more basic growth during childhood and the pubertal years (the Q-function growth by the QEPS model), resulting in taller adult height.

With a prevalence of around 1 in 667, Klinefelter syndrome (KS) (47, XXY) is the most frequent sex chromosome abnormality in males [1]. In the original description of KS from 1942, increased height was 1 of the classic phenotypes of individuals with KS together with eunuchoid body proportions, gynecomastia, small testes, elevated urinary gonadotropins, impaired body hair, and female fat distribution [2]. Increased height may be attributed to factors such as overexpression of the *SHOX* gene, which is located in the pseudoautosomal region of the sex chromosomes; variations in CAG repeat length in the androgen receptor gene; and the presence of insufficient sex steroid levels to initiate epiphyseal fusion [3-6]. Although detailed characterization of growth phases in KS has not been performed previously, it has been observed that birth length is typically normal in boys with KS but that growth accelerates during childhood and puberty, ultimately resulting in an adult height approximately 4 to 6 cm above the population mean and surpassing predictions based on parental height [4, 5, 7-13].

KS is highly underdiagnosed with an estimated 62% to 75% of males affected remaining undiagnosed [1, 14]. Importantly, KS is mainly diagnosed during infertility evaluations in adulthood, and a diagnosis before puberty is rare, representing only about 10% of all diagnoses of KS [1]. If KS is diagnosed before or during puberty, it is common to start with testosterone replacement therapy (TRT) during puberty. A detailed description and analysis of growth may help physicians identify potential cases of KS by recognizing specific growth trajectories and patterns associated with the condition.

The quadratic-exponential-pubertal-stop (QEPS) growth model describes growth from fetal life to adulthood and can be used to analyze growth patterns in a detailed way using four mathematical functions: a quadratic function (Q) representing basic continuous growth from early fetal life until the end of the growth period when all growth is stopped, which is represented by a stop function (S), on top of which 2 growth functions are added: the exponential function (E) that is specific for early life growth and the pubertal function (P) that is specific for pubertal growth [15-17] (Fig. 1). This model allows the user to look in detail at the different growth phases, including separating the contributions of ongoing basic growth (QES function-related) and puberty-specific growth (P function-related) during adolescence. It also allows individualization based on the timing and pattern of the pubertal growth spurt by using measures for age at the onset, middle, and end of puberty and provides height both in cm and in SD scores (SDS) with individual confidence intervals. The validated QEPS model [15-17] has been used to develop references based on chronological age and aligned on age at the onset of pubertal growth [18, 19]. Thus, the QEPS model provides a tool to describe, analyze, and compare growth in different populations during infancy, childhood, and adolescence. To date, the model has been used to explore secular growth trends and to characterize growth patterns in children with obesity and congenital adrenal hyperplasia [18-23].

**Figure 1.**
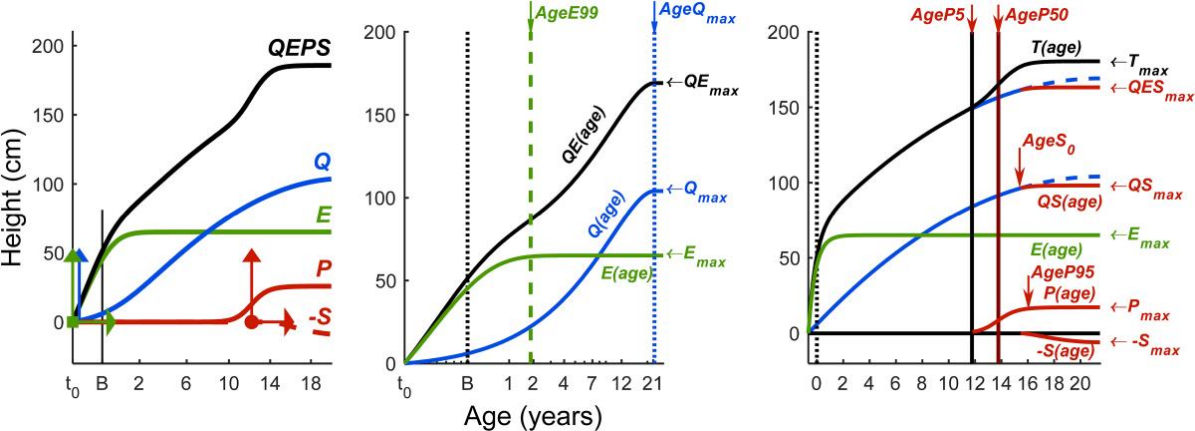
QEPS model with key parameters. *Left panel:* The 4 mathematical functions of the QEPS model that are combined to describe total gain in height (T) from fetal life to adulthood: Quadratic (Q), Exponential (E), Pubertal (P), and Stop (S). B = birth, marked with a vertical line. t_0_ = about 6 weeks after conception. Age scale below 3 years is stretched out. The QEPS model is fitted with 6 parameters. The 3 vertical arrows indicate the fitted individual height-scale parameters, from left to right *E_heightscale_*, *Q_heightscale_*, and *P_heightscale_*. The 2 horizontal arrows indicate the time-scale parameters, from left to right *E_timescale_* and *P_timescale_*, and the sixth parameter is the location of mid-puberty, *AgeP50*, indicated with a dot. *Middle panel:* QE model for prepubertal height, *QE(age)* = *E(age)* + *Q(age)*, *QE_max_ = E_max_* + *Q_max_*. *AgeE99* marks the age where 99% of *E_max_* is reached. *AgeQ_max_* marks the age when the maximum of Q-function growth is reached: for girls at 19.1 years and for boys at 21.5 years. Age is shown in logarithmic scale. *Right panel:* QEPS model for total height, *T(age)* = *E(age)* + *QS(age)* + *P(age)*, with *QS(age)* = *Q(age)*—*S(age)*. *T_max_ = E_max_* + *QS_max_ + P_max_*. *AgeP5* and *AgeP50* mark the ages where 5% and 50% of *P_max_* are reached; *AgeS_0_* marks the age where the S function is starting. *AgeP95* marks the age where 95% of *P_max_* is reached. Age is shown in linear scale. Abbreviation: QEPS, quadratic-exponential-pubertal-stop growth.

This study aimed to use the QEPS growth model to analyze in detail the spontaneous growth patterns of boys with KS during infancy and childhood. In addition, we aimed to evaluate puberty-specific growth in adolescents with KS who received TRT during puberty, as well as to characterize the adult height achieved in relation to parental heights and to a community-based healthy reference population: the GrowUp 1974 Gothenburg cohort [16, 17, 24, 25].

## Methods

### Study Populations

#### Patients with KS

Patients with nonmosaic 47,XXY KS who were followed longitudinally with measurements of height at the Department of Growth and Reproduction, Rigshospitalet, Copenhagen, Denmark were eligible for inclusion in this retrospective tertiary center study. Among the 96 eligible patients, 60 had sufficient height measurements from birth, infancy, and childhood to evaluate early growth using the QEPS model. After excluding individuals with excessively long intervals between measurements, which resulted in large confidence intervals (Table S1 [26]), the final study population included 55 boys. Thirty of these boys were diagnosed with KS prenatally whereas 20 boys were diagnosed during childhood due to delayed development, psychosocial challenges, cryptorchidism, or excessive growth. The reason for diagnosis was not available in 5 boys. A subgroup of 35 boys had data available throughout puberty to adult height; 34 of these boys received TRT.

The patients with KS were born between 1973 and 2011. Information on gestational age (GA) was not available for 16 of the 55 boys. However, none were considered small for a term baby based on birth weight. Therefore, these boys were included in the analysis, and data corresponding to 40 weeks of GA was applied in the model. None of the patients had other medical conditions or were on medications that are known to affect growth other than testosterone. Some data including heights from this population have been published previously [4, 11, 27].

#### Reference population

A subgroup of healthy boys from the GrowUp_1974_Gothenburg cohort served as the reference population. This cohort consisted of boys born in Sweden at full term (GA: 37-42 weeks) with longitudinal growth data available from birth to adult height (n = 1174). GA for boys in the GrowUp_1974_Gothenburg cohort was estimated based on data from the last menstrual period as documented in the medical birth record. The study populations and selection procedures have been described in detail elsewhere [16].

### Growth Data Anthropometry

#### Patients with KS

Infants were measured to the nearest 0.5 cm until 2 years of age using a baby length measuring mat. From the age of 2 years, standing height was measured to the nearest 0.1 cm using a wall-mounted Harpenden stadiometer. For completion of growth data, measurements of length/height made at birth and at routine child examinations by the general practitioner were included. Data on birth length were available in 51 boys. Following their diagnosis of KS, the boys were followed at the Department of Growth and Reproduction, Rigshospitalet, Copenhagen, Denmark. Regular assessments of pubertal maturation were conducted, including Tanner genital staging and testicular volume measurement by palpation using a Prader orchidometer.

#### Reference population

Length/height was measured at well-baby clinics, child healthcare centers, and school. A specially trained team measured the children aged 17 to 18 years in their 11th school year. Height was measured using a calibrated Harpenden stadiometer. Individuals who were still growing were followed with additional measurements by the study team until adult height was attained, 0.5 cm/previous year [17, 25].

### TRT

A total of 48 patients received TRT, with treatment initiated at a median age of 13.2 years (Table 1). Patients receiving TRT were typically started on oral testosterone undecanoate (different brands according to availability and period: Restandol [Organon, Oss, The Netherlands], Andriol [Orifarm, Hostivice, Czech Republic], or Testosterone “Paranova” [Paranova Danmark A/S, Herlev, Denmark]), and most were transferred to transdermal testosterone (Tostran 2% [Kyowa Kirin, Holland]) with individualized dose titration according to serum concentrations of testosterone and LH, and in late puberty sometimes to intramuscular testosterone undecanoate (Nebido). The starting dose of oral testosterone undecanoate was typically 40 mg daily, increasing to 80 mg twice daily. Treatment was initiated after the clinical onset of puberty (testicular volume >3 mL) if the patient presented with symptoms of testosterone deficiency (eg, gynecomastia, fatigue, low muscle tone, and obesity) or if the serum concentration of LH exceeded +2 SD according to age- and sex-adjusted references. Some patients were diagnosed with KS during late puberty, and TRT was therefore initiated after the age of 14 years (n = 10).

**Table 1.** Basic characteristics of KS vs the reference group

	Descriptive data			Test between groups	
Variable	Klinefelter n = 55 (prepubertal)	Klinefelter n = 35 (pubertal)	1974 reference n = 1174	*P*-value for Klinefelter prepub vs 1974 reference	*P*-value for Klinefelter pub vs 1974 reference
BW (g)	3356 ± 639 3450 (1850-4900) n = 53	3365 ± 607 3450 (2050-4900) n = 33	3517 ± 486 3520 (1810-5420) n = 1174	.08	.16
BW_SDS_	−0.75 ± 1.38 −0.94 (−3.08-2.82) n = 38	−0.60 ± 1.47 −0.81 (−2.92-2.82) n = 24	−0.60 ± 1.12 −0.50 (−6.67-2.58) n = 1174	.50	1.00
BL (cm)	50.8 ± 3.2 51.0 (42.0-58.0) n = 51	51.0 ± 2.8 51.0 (43.0-58.0) n = 32	50.5 ± 2.1 51.0 (41.0-60.0) n = 1174	.52	.36
BL_SDS_	−0.43 ± 1.52 −0.78 (−3.02-3.67) n = 36	−0.20 ± 1.43 −0.53 (−2.33-3.67) n = 23	0.05 ± 1.01 0.01 (−3.52-3.14) n = 1174	.07	.40
Mothers' height (cm)	167.9 ± 6.3 169.3 (152.1-178.1) n = 46	167.0 ± 6.0 166.2 (157.0-178.1) n = 29	166.8 ± 6.2 167.0 (148.0-189.0) n = 892	.24	.83
Fathers' height (cm)	179.2 ± 7.9 180.1 (164.6-196.0) n = 46	179.3 ± 7.4 180.2 (164.8-193.6) n = 29	179.5 ± 6.7 180.0 (160.0-202.0) n = 898	.76	.85
Age at testosterone treatment (years)	13.6 ± 2.1 13.2 (11.0-22.5) n = 48	13.4 ± 1.8 13.3 (11.0-18.2) n = 34			

Data are presented as mean ± SD, SD score, median (range) and number of observations, or number (percentage). For tests between 2 groups with respect to skewed continuous variables, the Mann-Whitney U-test was used and for normally distributed data, the 2-sample *t*-test was used.

Abbreviations: BL, birth length; BW, birth weight.

### Definitions of Non-QEPS Growth Variables

#### Parental height

For boys with KS, parental heights were available for 45 boys. Data on parental heights were mainly obtained by measurement in the outpatient clinic, but in some cases, they were reported. For the reference population, maternal height measurements for the study participants were obtained from the medical birth record, whereas data on paternal height measurements came from questionnaires answered by the parents or the study participants in the high schools or from records at child healthcare centers.

#### Adult height

Adult height was defined as the height measured when the growth rate had declined to less than 0.5 cm per year.

### Diffmph-tmaxsds

The calculated difference between the individual´s *T_maxSDS_* (total height by the QEPS model) and the height of the parents (MPH_SDS_) is a measure of the intrafamilial height difference (DiffMPH-*T_maxSDS_*).

### The QEPS Growth Model

The QEPS model was used to describe individual patterns of growth from birth to adult height. Growth in height was modeled by a Quadratic (Q) function, a negative Exponential (E) function, a specific Pubertal (P) function, and a Stop (S) function. For more information, see Fig. 1 and previous publications [15-18]. For all QEPS variables (except *E_timescale_*), the height is given in centimeters and the individual's age in years. The SDSs for the QEPS variables were computed from the GrowUp_1990_Gothenburg reference population [21].

### Definitions of QEPS Growth Variables

#### Basic total growth

The ongoing basic growth during the prepubertal period, starting soon after conception, is described by the Q- and E-functions of the model (*Q_max_, E_max,_* and *E_timescale_*), and later also by the *S*-function, which starts at *AgeS_0_* (Fig. 1).

#### Prepubertal growth: Early life, fetal/infancy growth period

This covers the period from approximately 6 weeks after conception until the end of infancy (*AgeE99*). Fetal growth ends at birth (*QE_birth_*) when it is assessed as length/weight according to GA. The infancy growth period starts at birth and ends at *AgeE99*, by which time 99% of the E function's amplitude has been reached.

#### Childhood growth period

This covers the period from the end of infancy (*AgeE99*) to the onset of pubertal growth (*AgeP5*). Childhood growth was calculated vs *Q_max_, E_max_* and *QE_max_*.

Onset of the pubertal growth period was defined at the point where 5% of the specific pubertal growth function P was reached, *AgeP5;* mid-pubertal growth with *AgeP50* (where 50% of pubertal growth was attained); and end when 95% of the pubertal growth was attained as *AgeP95* [17]. With the different pubertal QEPS growth estimates, the duration of pubertal growth can be described. The QEPS growth model can separate growth into 2 components: growth that is specific to puberty (P function growth, *P_max_*) and the basic growth, which continues during puberty (QES function growth). In this way, the total gain in height during puberty (pubgain) can be described for T, QES, and P. Age at peak height velocity (PHV) can be calculated as *AgeTPHV* from the total curve.

### Statistical Analysis

QEPS variables and most of the related figures were constructed in Matlab as previously described [16]. Mathselect used to evaluate quality of growth data [17]. Student's *t*-test was used to analyze variables with a normal distribution. For variables with skewed distribution, the nonparametric Mann–Witney U-test was used. A *P*-value < .05 was considered statistically significant. SAS software version 9.4 (SAS Institute Inc., Cary, NC, USA) was used for handling basic data and for constructing some of the figures.

## Ethical Approval

Patients with KS and their parents (if the patient was below 18 years of age) gave informed consent for clinical follow-up. Data from routine clinical visits were obtained from patient records. Access to patient data was approved by the Danish Patient Safety Authority (no. 3-3013-1376/1/) and the Team for Medical Records Research, Centre for Health, the Capital Region of Denmark (no. R-22031906). The study was registered at the Capital Region of Denmark according to the European Union GDPR (no. P-2022-364), and ethical approval for analyzing data in Sweden was obtained from the Swedish national ethic committee (EPM 2022-07095-01).

For subjects in the GrowUp_1974_Gothenburg cohort, ethical approval was obtained from the Regional Ethics Review Board in Gothenburg (Ad 91-92/131-93 and 444-08 T062-09), and all participants or their legal guardians gave informed consent.

## Results

### Description of the Study Population of Boys with KS Compared with the Reference Population

Characteristics of the boys with KS (total group and subgroup reaching adult height) are presented and compared with the reference group in Table 1. No difference in body weight, body length, or parental heights was found when comparing the total group of boys with KS with the reference population.

### Prepubertal Period: Tempo and Growth

#### Fetal/infancy growth

**Figure 2.**
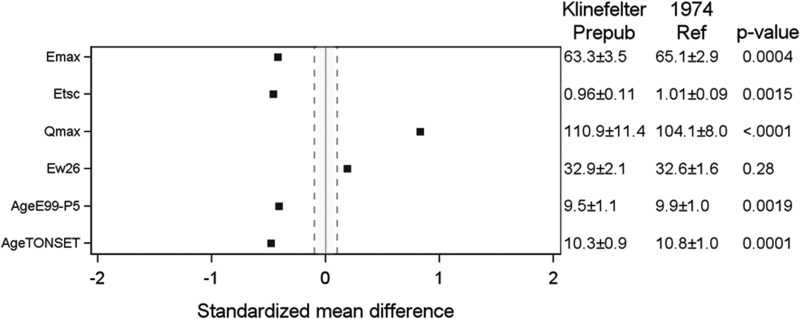
Standardized mean difference for QEPS variables for the KS prepubertal group vs reference. *E_max_* = gain in adult height in cm due to the E-function growth, mainly representing fetal and infant growth. *E_timescale_* = time-scale parameter for the E function; a shorter *E_timescale_* means that the E-max is reached earlier. *Q_max_* = gain in adult height in cm due to Q-function growth, mainly representing childhood growth. *P_max_* = pubertal gain in adult height in cm due to the specific P-function growth. *AgeE99* = age at which 99% of the E-function growth is reached. *AgeE99-P5* = the growth period of childhood (from end of infancy to start of the pubertal growth). Abbreviations: KS, Klinefelter syndrome; QEPS, quadratic-exponential-pubertal-stop growth.

QEPS model estimates for growth during fetal life and infancy for boys with KS vs the reference population are presented separately for the total group (n = 55) and the subgroup with available data in all age periods including pubertal growth (n = 35) data in Table 2 and Fig. 2. The boys with KS experienced less growth during the infancy period than boys in the reference population as reflected by a lower *E_max_*, 63.3 and 63.4 (total group and subgroup, respectively) vs 65.1 cm (*P* < .001 for both) and shorter *E_timescale_* 0.96 and 0.96 (total group and subgroup, respectively) vs 1.01 (*P* = .002 and .001, respectively); their infancy growth period was also shorter, ending at an earlier age than in the reference population (*AgeE99*, 1.76 vs 1.88 years, respectively) (*P* = .002 and .001, respectively).

**Table 2.** QEPS variables (nonpubertal) for Klinefelter vs the reference group

Variable	Klinefelter n = 55	1974 reference n = 1174	*P*-value for Klinefelter prepub vs 1974 reference
E_max_ (cm)	63.3 ± 3.5 63.2 (57.3-72.0) n = 55	65.1 ± 2.9 65.1 (56.6-74.8) n = 1174	.0004
E_tsc_	0.96 ± 0.11 0.95 (0.72-1.28) n = 55	1.01 ± 0.09 1.00 (0.69-1.30) n = 1174	.0015
AgeE99 (years)	1.75 ± 0.29 1.73 (1.16-2.56) n = 55	1.88 ± 0.22 1.87 (1.08-2.61) n = 1174	.0015
Q_max_ (cm)	110.9 ± 11.4 110.7 (83.1-134.5) n = 55	104.1 ± 8.0 103.9 (73.7-135.3) n = 1174	<.0001
Age_EE99-P5_ (years)	9.5 ± 1.1 9.4 (6.9-11.6) n = 55	9.9 ± 1.0 9.9 (6.5-12.9) n = 1174	0.0019

Variable	Klinefelter n = 35	1974 reference n = 1174	*P*-value for Klinefelter pub vs 1974 reference
E_max_ (cm)	63.4 ± 3.3 63.2 (57.5-72.0) n = 35	65.1 ± 2.9 65.1 (56.6-74.8) n = 1174	.0006
E_tsc_	0.96 ± 0.10 0.96 (0.72-1.18) n = 35	1.01 ± 0.09 1.00 (0.69-1.30) n = 1174	.0012
AgeE99 (years)	1.76 ± 0.26 1.76 (1.16-2.31) n = 35	1.88 ± 0.22 1.87 (1.08-2.61) n = 1174	.0012
Q_max_ (cm)	111.5 ± 11.9 112.6 (83.1-134.5) n = 35	104.1 ± 8.0 103.9 (73.7-135.3) n = 1174	.0008
AgeE_E99-P5_ (years)	9.6 ± 1.1 9.5 (6.9-11.6) n = 35	9.9 ± 1.0 9.9 (6.5-12.9) n = 1174	.032

Data are presented as mean ± SD, median (range) and number of observations, or number (percentage). *E_max_* = gain in adult height in cm due to the E-function growth, mainly representing fetal and infant growth. *E_tsc_ = E_timescale_* time-scale parameter for the E function; a shorter *E_timescale_* means that the *E_max_ i*s reached earlier. *AgeE99* = age at which 99% of the E-function growth is reached. Q_max_ = gain in adult height in cm due to Q-function growth, mainly representing childhood growth. *AgeE_99-P5_* the growth period of childhood (from end of infancy to start of the pubertal growth). For tests between 2 groups with respect to normally distributed data, a 2-sample *t*-test was used.

Abbreviation: QEPS, quadratic-exponential-pubertal-stop.

#### Childhood growth

Boys with KS experienced more Q-function growth than the reference population, with *Q_max_* 110.9 and 111.5 (total group and subgroup, respectively) vs 104.1 cm (*P* < 0001 and .001, respectively). The duration of the childhood growth period (*AgeE99* to *AgeP5*) was0.4 years shorter in boys with KS compared with the reference group: 9.5 and 9.6 (total group and subgroup, respectively) vs 9.9 years (*P* = .002 and .03, respectively).

### Pubertal Period: Tempo, Timing, and Growth

#### Pubertal timing and tempo

**Figure 3.**
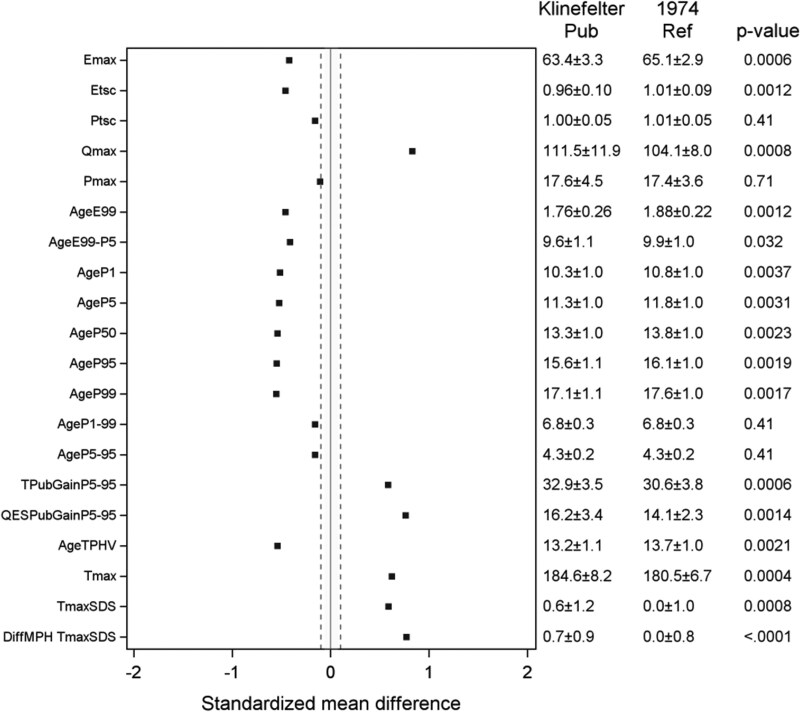
Standardized mean difference for QEPS variables for the KS pubertal group vs reference. *E_max_* = gain in adult height in cm due to the E-function growth, mainly representing fetal and infant growth. *E_timescale_* = time-scale parameter for the E-function; a shorter *E_timescale_* means that the E-max is reached earlier. *P_timescale_* = the pubertal timescale parameter; a shorter *P_timescale_* means that the duration of pubertal growth is faster. *Q_max_* = gain in adult height in cm due to Q-function growth, mainly representing childhood growth. *AgeE99* =age at which 99% of the E-function growth is reached. *AgeE_99-P5_* = the growth period of childhood (from end of infancy to start of the pubertal growth). *AgeP1* = age at which 1% of the P-function growth is reached, *AgeP5* to *AgeP99* is the age where the corresponding percentage of the P-function growth is reached; the *AgeP_1-99_* and *AgeP_5-95_* is the duration of pubertal growth between these timepoints. *TPubGainP_5-95_* shows the total gain in height (cm) during this timepoints, and *QEPSPubGainP_5-95_* shows how much of this gain that is due to the continuous QES function*. AgeTPHV* is the age at peak height velocity from the total growth curve T. *T_max_* is the modeled adult height, the maximal function, ie, height in cm. DiffMPH-*T_maxSDS_* is the calculated difference between the individuals *T_maxSDS_* (height), and the height of the parents (MPHSDS) is a measure of the intrafamilial height difference. Abbreviations: KS, Klinefelter syndrome; MPHSDS, mid-parental height SD score; QEPS, quadratic-exponential-pubertal-stop growth.

Boys with KS reached pubertal growth milestones earlier with an earlier onset of pubertal growth (*Age_P5_* 11.3 vs 11.8 years [*P* = .003]) and an earlier end of pubertal growth (*AgeP95* 15.6 vs 16.1 years [*P* = .002]) as compared with the reference population with a similar duration (Table 3 and Fig. 3).

**Table 3.** QEPS variables (pubertal and adult height) for Klinefelter vs the reference group

Variable	Klinefelter n = 35	1974 reference n = 1174	*P*-value for Klinefelter pub vs 1974 reference
P_tsc_	1.00 ± 0.05 1.00 (0.91-1.09)	1.01 ± 0.05 1.01 (0.76-1.18)	.41
P_max_ (cm)	17.6 ± 4.5 17.2 (7.9-29.5)	17.4 ± 3.6 17.5 (4.1-28.9)	.71
AgeP_1_ (years)	10.3 ± 1.0 10.4 (8.1-12.3)	10.8 ± 1.0 10.8 (7.5-13.9)	.0037
AgeP_5_ (years)	11.3 ± 1.0 11.4 (9.2-13.4)	11.8 ± 1.0 11.8 (8.6-14.9)	.0031
AgeP_50_ (years)	13.3 ± 1.0 13.4 (11.3-15.5)	13.8 ± 1.0 13.8 (10.7-16.9)	.0023
AgeTPHV (years)	13.2 ± 1.1 13.3 (11.1-15.4)	13.7 ± 1.0 13.7 (10.6-16.8)	.0021
AgeP_95_ (years)	15.6 ± 1.1 15.6 (13.6-18.0)	16.1 ± 1.0 16.1 (13.1-19.3)	.0019
AgeP_99_ (years)	17.1 ± 1.1 17.1 (15.0-19.6)	17.6 ± 1.0 17.6 (14.6-20.7)	.0017
AgeP_P1-99_ (years)	6.8 ± 0.3 6.7 (6.2-7.4)	6.8 ± 0.3 6.8 (5.1-8.0)	.41
AgeP_P5-95_ (years)	4.3 ± 0.2 4.3 (3.9-4.7)	4.3 ± 0.2 4.3 (3.2-5.0)	.41
TPubGain_P5-95_ (cm)	32.9 ± 3.5 33.3 (26.8-44.3)	30.6 ± 3.8 30.6 (17.6-42.2)	.0006
QESPubGain_P5-95_ (cm)	16.2 ± 3.4 16.2 (10.0-24.2)	14.1 ± 2.3 14.1 (7.5-23.2)	.0014
T_max_ (cm)	184.6 ± 8.2 184.1 (170.4-205.2)	180.5 ± 6.7 180.3 (157.3-201.1)	.0004
T_max_SDS	0.6 ± 1.2 0.5 (−1.5-3.7)	0.0 ± 1.0 −0.0 (−3.5-3.0)	.0008
DiffMPH T_max_SDS	0.7 ± 0.9 0.8 (−1.0-3.1) n = 29	0.0 ± 0.8 0.0 (−2.3-3.1) n = 872	<.0001

Data are presented as mean ± SD, median (range) and number of observations, or number (percentage). *P_timescale_* is the pubertal timescale parameter; a shorter *P_timescale_* means that the duration of pubertal growth is faster. *AgeP1* is the age at which 1% of the P-function growth is reached. *AgeP5* to *AgeP99* is the age where the corresponding percentage of the P-function growth is reached; the *AgeP_1-99_* and *AgeP_5-95_* is the duration of pubertal growth between this timepoints. TPubGainP_5-95_ shows the total gain in height (cm) during this timepoints and *QEPSPubGainP_5-95_* shows how much of this gain that is due to the continuous QES function. *AgeTPHV* is the age at peak height velocity from the total growth curve T. *T_max_* is the modeled adult height, the maximal function, ie, height in cm*. DiffMPH-T_maxSDS_* is the calculated difference between the individual´s *T_max_SDS* (height) and the height of the parents (mid-parental height SD score) is a measure of the intrafamilial height difference. For tests between 2 groups with respect to normally distributed data, a 2-sample *t*-test was used.

Abbreviation: QEPS, quadratic-exponential-pubertal-stop.

#### Pubertal height gain

**Figure 4.**
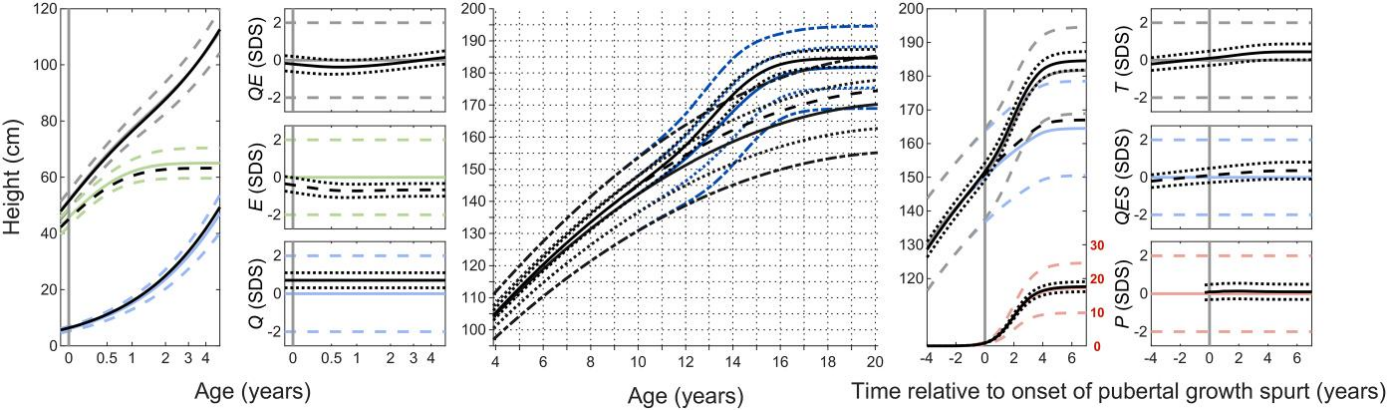
Longitudinal height trajectories with QEPS functions for the KS study group related to the reference. Left panel: Prepubertal group n = 55; mean height in cm vs age 0 to 4 years for QE (top, black solid), E (middle, black dashed), and Q (bottom) function values and corresponding mean±95% CI function values in SDS. Middle panel: Pubertal group n = 35, mean height in cm vs age 4 to 20 years for QEPS (black solid, ±95% CI black dotted) and mean QE (black dashed) function values. Right panel: Pubertal group n = 35; mean height in cm vs puberty adjusted age for QEPS (top, black solid, ±95% CI black dotted), QES (middle, black dashed), and P (bottom, ±95% CI black dotted) function values and corresponding function values in SDS. Abbreviations: CI, confidence interval; KS, Klinefelter syndrome; QEPS, quadratic-exponential-pubertal-stop; SDS, SD score.

**Figure 5.**
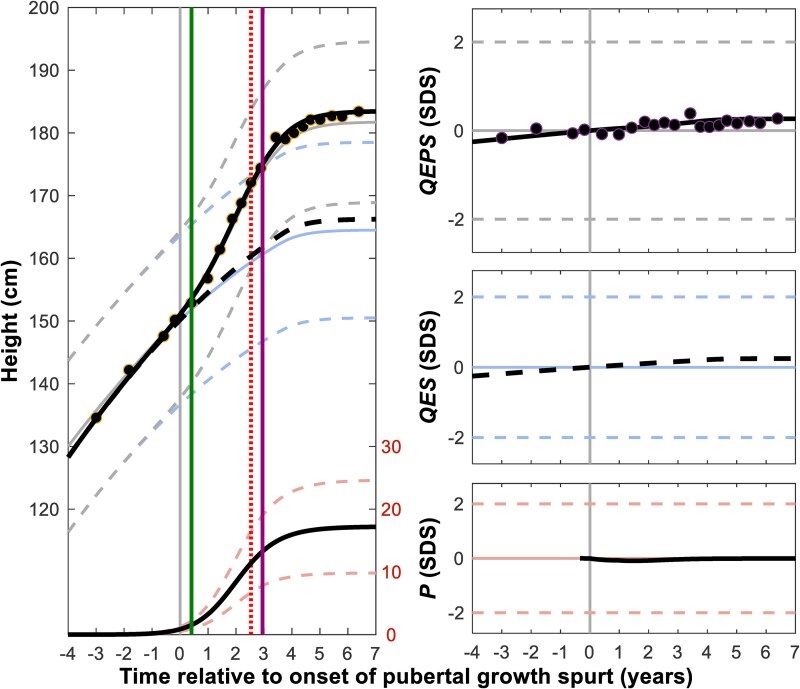
Individual height trajectories around puberty for a typical KS patient. Pubertal growth for a typical individual boy with KS vs the P-age reference aligned for *AgeP5* as onset of puberty (grey line, first line from the left), revealing total QEPS, basic QES, and specific pubertal growth. Vertical lines indicate the time of pubertal onset as defined by testicular volume >3 mL (green line, second line from the left) and by the time where the serum concentration of LH exceeded +2 SDS (violet line, fourth line from the left), as well as for the age at the start of testosterone treatment (dotted red line, third line from the left). Abbreviations: KS, Klinefelter syndrome; QEPS, quadratic-exponential-pubertal-stop.

Boys with KS experienced more total growth during puberty compared with the reference population, with a *Tpubgain_P5-P5_* of 32.9 vs 30.6 cm (*P* < .001), due to more *QESpubgain* 16.2 vs 14.1 cm (*P* = .001) (Table 3, Figs. 3 and  4). The specific pubertal growth, as *P_pubgain_* and *P_max_* 17.6 vs 17.4 cm (*P* = .71), did not differ in these boys with KS vs the reference population. A significant association between age at starting TRT and onset of pubertal growth as *AgeP5* was found. Pubertal growth for a typical individual boy is shown in Fig. 5 vs the P age reference aligned for *AgeP5* as onset of pubertal growth.

### Adult Height

**Figure 6.**
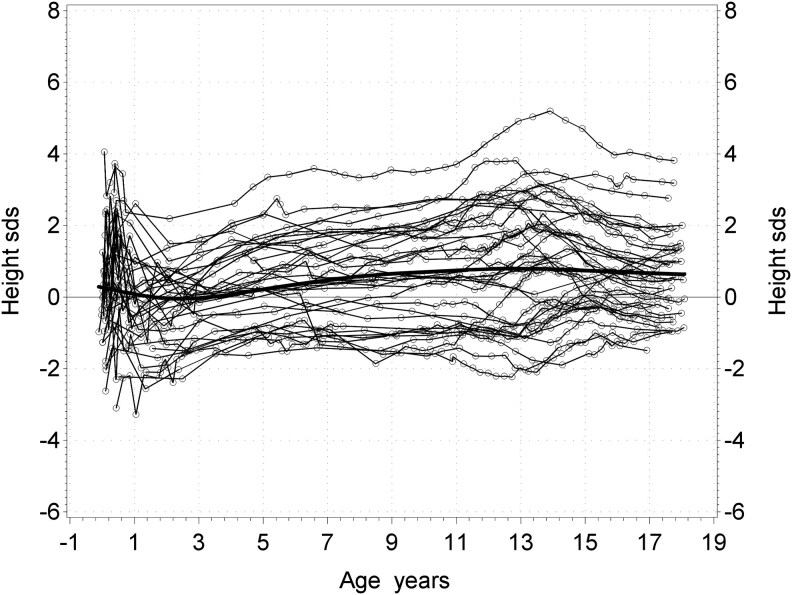
Longitudinal height_SDS_ from birth to adult height for all individuals in the KS pubertal group. Abbreviation: KS, Klinefelter syndrome.

As seen in Fig. 3 and Table 3, at adult height, boys with KS were taller than boys in the reference group, with an adult height (*T_max_*) of 184.6 vs 180.5 cm, corresponding to a height_SDS_ of +0.6, which gave a DiffMPH-*T_maxSDS_* of +0.7 (*P* < .001). All individual growth trajectories are visualized in Fig. 6 with traditional SDS lines of height for the 35 KS patients with sufficient longitudinal data in all growth periods.

## Discussion

In this comprehensive analysis of growth in boys with KS, using the QEPS model from fetal life through infancy, childhood, and puberty to adult height, we identified specific growth pattern changes when compared to a healthy reference population. Boys with KS experienced less growth during early life but more growth during childhood and puberty, resulting in an increased adult height.

The QEPS model characterized spontaneous prepubertal growth patterns in boys with KS, showing that the boys with KS experienced less growth during early life (infancy growth), obtained during a shorter period. However, boys with KS experienced more basic growth, resulting in more growth during childhood compared with boys in the reference population. Also, during pubertal years, boys with KS grew more than the reference population because of more basic growth during this period, while the quantity of specific pubertal growth did not differ between the 2 groups. The boys with KS both entered and ended pubertal growth at an earlier age but with similar duration as the reference population. Consequently, boys with KS were taller as adults both relative to the reference group and to their parental heights. Thirty-four of the 35 boys reaching their final height received TRT during puberty.

### Benefit of Using a Mathematical Growth Model

We here use the QEPS growth model as a tool to separate growth into different functions, allowing a more detailed exploration of growth patterns in boys with KS. Specifically, we use the E-function to examine early life growth, and the P-function to explore pubertal growth [16, 17]. Both describe growth in parallel to the ongoing basic Q-function growth from fetal life to adulthood. We are using and interpreting mathematical information of the functions in our understanding that growth during different periods is regulated differently, and thereby the knowledge obtained can be helpful in further understanding underlying regulations of physiology of growth in future studies. To our knowledge, this is the first study using a growth model to characterize growth patterns in patients with KS.

We found no significant differences in birth weight, birth length, or parental heights between the groups. This is in line with most previous research, whereas a smaller size at birth also has been described [4, 28-31].

During infancy, boys with KS exhibited less growth compared to the reference population, as indicated by a lower Emax, and a shorter infancy growth period. In line with previous studies describing relatively tall stature already in childhood [8, 9, 31, 32], we found more Q-function growth during childhood, resulting in a taller stature compared with the reference population. Interestingly, the childhood growth period was 0.4 years shorter than the reference.

Boys with KS reached pubertal growth milestones earlier, with an earlier onset (*AgeP5*) and end (*AgeP95*) of pubertal growth compared to the reference population but with a similar duration of the period. The boys with KS experienced more total growth during puberty due to increased Q-function. Specific pubertal growth did not differ significantly. The amount and pattern of puberty-specific growth in the 35 boys of whom 34 received TRT (either oral or transdermal) was comparable to that observed in the reference population. The boys with KS started TRT at a median age of 13.3 (range 11.0-18.2) years, with no association between age at starting TRT and *P_max_*. Importantly, mean age at starting TRT was approximately 2 years later than the age of onset of the pubertal growth (11.4 [range 9.2-13.4] years), and TRT therefore does not seem to explain the earlier onset of pubertal growth in the boys with KS.

It is a common misconception in general pediatrics that KS is associated with delayed puberty [32]. Previous research has found onset of puberty in boys with KS at similar ages as the background population [31-35]. Our finding that the pubertal growth spurt started on average 0.4 years earlier in boys with KS compared with the reference population may be considered surprising. Theoretically, it could be due to a difference between the Danish and Swedish populations. However, no previous studies have shown any clear differences in the timing of pubertal growth or secular trend between Denmark and Sweden, making this explanation less likely [36]. The difference may be related to methodological aspects; the QEPS model very precisely calculates the onset of pubertal growth, and it is possible that previous studies, without using a growth model, would not detect a difference of some months in the timing of the pubertal growth spurt. Most previous studies have evaluated puberty related to secondary sexual characteristics, not analyzing pubertal growth. A third possible explanation for earlier pubertal growth may be due to hormonal changes or a different sensitivity to hormones in early puberty for boys with KS. IGF-I and IGFBP-3 concentrations are normal [4], but FSH and LH levels usually increase after pubertal onset [27, 32, 34] and have been shown to exceed the level of healthy controls at Tanner stage 3 [34]. This hormonal profile is generally indicative of primary testicular failure; however, the elevated levels of FSH and LH may paradoxically contribute to an earlier onset of pubertal growth. The first 6 to 12 months of puberty is the pubertal growth spurt often not seen in healthy boys. The increased gonadotropin levels could potentially stimulate the testes to produce testosterone earlier than expected. This early testosterone production, although insufficient for complete pubertal development, could theoretically contribute to some months earlier onset of pubertal growth compared to the reference population. A fourth possible explanation for the earlier onset of pubertal growth may be genetic, attributed to the presence of an extra X chromosome. The *SHOX* gene, located in the PAR1 region of the X chromosome, escapes inactivation, resulting in 3 active copies in boys with KS. This increased gene dosage has been directly linked to taller stature [5].

### Strengths and Limitations

The major strength of this study is the well-characterized cohort of boys and adolescents with KS followed at a single tertiary center and the inclusion of a large cohort of 1174 healthy controls.

Comparing the Danish cohort of patients with KS with a Swedish reference population may be considered as a weakness of the study design. However, the parental heights of the 2 different populations were very similar and with a difference (not significant) of 1.1 cm between the groups for mothers and 0.3 cm for fathers. Additionally, in a comparison between Danish and Swedish reference curves, final heights were similar [37].

A strength in our study compared to other studies on growth patterns in patients with KS was the use of a growth model. The QEPS model can describe and analyze the different growth periods, including a very precise and standardized method for evaluation of growth.

Another strength compared to other studies is the full pattern of growth from birth to achieved adult height where other studies write about “near final height “or “near adult height,” making their analyses and conclusions less precise. Most previous studies of growth in patients with KS have analyzed cross-sectional data or have been based on a limited numbers of individuals without control groups [34]. The risk of ascertainment bias poses a challenge when studying growth in boys with KS, as the majority of affected individuals are diagnosed late or remain undiagnosed [1, 14]. In the present cohort, however, 60% were diagnosed prenatally, which likely contributes to a more representative sample encompassing the full phenotypic spectrum.

A potential limitation is that the reference population was born in the mid-1970s, whereas the boys with KS were born between 1974 and 2011. However, we have previously shown that final height in healthy Danish boys born between 1987 and 2002 was similar to that of the Swedish boys born in 1973 to 1975 [37], and the secular increase in height appears to have largely plateaued in the Nordic countries during the late 20th century. Another limitation is the retrospective nature of the study. Not all boys were followed through puberty to adult height. Some but not all boys started TRT during follow-up, and some started TRT in late puberty. The study was not designed to evaluate the effect of TRT on growth, and detailed information about dose titration was not available. Future prospective studies are needed to clearly identify potential influences of the timing and progression of puberty and the influence of TRT on the growth patterns in boys with KS. Studies combining detailed information on both hormonal levels, pubertal development, and auxological data in late childhood/early puberty may add further insight of the findings of a slightly earlier pubertal growth for boys with KS found in our study.

## Conclusion

We present detailed mathematical modeling of growth patterns from birth to adult height for individuals with KS using the QEPS growth model. We found that individuals with KS had a different growth pattern as compared with a healthy reference population, with less and shorter growth in early/infant life and more basic growth during childhood and the pubertal years (the Q-function growth by the QEPS model), resulting in taller adult height.

## Data Availability

Data generated and/or analyzed during the current study are not publicly available but are available from the corresponding author on reasonable request.

## Abbreviations

DiffMPH-*T_maxSDS_*: calculated difference between the individual´s TmaxSD score (height) and the height of the parents (mid-parental height SD score), a measure of the intrafamilial height difference
GA: gestational age
KS: Klinefelter syndrome
MPH: mid-parental height
PHV: peak height velocity
QEPS: quadratic-exponential-pubertal-stop growth
SDS: SD score
TRT: testosterone replacement therapy

**QEPS Model-Derived Abbreviations:**
AgeE99: age at which 99% of the E-function growth is reached
AgeP5: age at which 5% of the P-function growth is reached
AgeP50: age at which 50% of the P-function growth is reached
AgeP95: age at which 95% of the P-function growth is reached
AgeP99: age at which 99% of the P-function growth is reached
AgeP_PHV_: age at peak height velocity of the P function
AgeT_PHV_: age at peak height velocity of the T function
E: negative exponential growth function of age *E(age)* in cm
E_max_: gain in adult height in cm due to the E-function growth
E_timescale_: time-scale parameter for the E-function
P: quadratic logistic function of age describing the pubertal growth spurt *P(age)* in cm
P%: percentage of achieved specific pubertal growth at time given
P_max_: pubertal gain in adult height in cm due to the specific P-function growth
P_pubgain_: *P_max_ –P(AgeP5*), height in cm due to the P function during the period P5-P100
Q: quadratic growth function of age Q (age) in cm
QES: basic height function in cm = total height T function minus specific puberty P function
QES(age): *T(age) – P(age)* *=* *QEPS(age) – P(age)*
QESpubgain: *QES_max_ – QES(AgeP5*), height gain in cm due to the basic QES function during the period P5-P100
Q_max_: gain in adult height in cm due to Q-function growth
S: stop function *S (age)* in cm, stopping the Q function at the end of growth
T: total height function in cm; *T(age)* = *QEPS(age)* *=* *Q(age)* *+* *E(age)* *+* *P(age) – S(age)*
T_AgeP5_: function value of total height function T at P5 in cm
T*_AgeP50_*: function value of total height function T at P50 in cm
T_max_: *QEPS_max_* , modeled total adult height in cm, *T_max_ = E_max_ + Q_max_ + P_max_ – S_max_*
T_max_AH: adult height in cm. It aligns with *T_max_*, except in instances where the measured height at the inception of the study surpasses *T_max_*; in such cases, the measured height is used
Tpubgain: *T_max_ – T(AgeP5),* total height in cm during the period P5-P100
